# Lactate Albumin Ratio Is Associated With Mortality in Patients With Moderate to Severe Traumatic Brain Injury

**DOI:** 10.3389/fneur.2022.662385

**Published:** 2022-04-01

**Authors:** Ruoran Wang, Min He, Fengyi Qu, Jing Zhang, Jianguo Xu

**Affiliations:** ^1^Department of Neurosurgery, West China Hospital, Sichuan University, Chengdu, China; ^2^Department of Critical Care Medicine, West China Hospital, Sichuan University, Chengdu, China; ^3^Department of Radiation Oncolygy, The First Affiliated Hospital of Xi'an Jiaotong University, Xi'an, China

**Keywords:** traumatic brain injury, lactate, albumin, lactate to albumin ratio, prognosis

## Abstract

**Background:**

Traumatic brain injury (TBI) is a serious public health issue all over the world. This study was designed to evaluate the prognostic value of lactate to albumin ratio (LAR) on patients with moderate to severe TBI.

**Methods:**

Clinical data of 273 moderate to severe TBI patients hospitalized in West China Hospital between May 2015 and January 2018 were collected. Multivariate logistic regression analyses were used to explore risk factors and construct a prognostic model of in-hospital mortality in this cohort. A receiver operating characteristic (ROC) curve was drawn to evaluate the discriminative ability of this model.

**Results:**

Non-survivors had higher LAR than survivors (1.09 vs. 0.53, *p* < 0.001). Results of multivariate logistic regression analysis showed that Glasgow Coma Scale (GCS; odds ratio [OR] = 0.743, *p* = 0.001), blood glucose (OR = 1.132, *p* = 0.005), LAR (OR = 1.698, *p* = 0.022), subdural hematoma (SDH; OR = 2.889, *p* = 0.006), intraparenchymal hemorrhage (IPH; OR = 2.395, *p* = 0.014), and diffuse axonal injury (DAI; OR = 2.183, *p* = 0.041) were independent risk factors of in-hospital mortality in included patients. These six factors were utilized to construct the prognostic model. The area under the ROC curve (AUC) values of single lactate, albumin, and LAR were 0.733 (95% Cl; 0.673–0.794), 0.740 (95% Cl; 0.683–0.797), and 0.780 (95% Cl; 0.725–0.835), respectively. The AUC value of the prognostic model was 0.857 (95%Cl; 0.812–0.901), which was higher than that of LAR (Z = 2.1250, *p* < 0.05).

**Conclusions:**

Lactate to albumin ratio is a readily available prognostic marker of moderate to severe TBI patients. A prognostic model incorporating LAR is beneficial for clinicians to evaluate possible progression and make treatment decisions in TBI patients.

## Introduction

Traumatic brain injury (TBI), defined as an alteration of brain function or other evidence of brain pathology due to external force, is a serious public health problem worldwide (1). Over the past decade, the prognosis of TBI has been improved significantly, attributable to better implementation of pre-admission treatments, rapid CT examination, and high-standard critical care measures (2). However, the continuously increased incidence rate of TBI makes it still a serious public health issue. Consequently, predicting the possible prognosis of patients in the early stage and then making suitable treatment strategies are significant to improve the outcomes of TBI patients. The serum lactate level, a widely acknowledged indicator of tissue hypoperfusion, has been confirmed associated with organ failure and mortality in many clinical settings, such as sepsis, trauma, and pediatric critical Illness (3–7). In addition, several studies have been conducted to explore the prognostic value of serum lactate level in TBI patients (8–10). Most of these studies showed that higher serum lactate was associated with worse injury severity and poor outcome in TBI patients. As an important metabolic marker of the whole body, serum lactate level is actually influenced by many factors, such as hepatic and renal function.

In order to stabilize and improve the predictive value of serum lactate, the lactate to albumin ratio (LAR), a new marker which synthetically combines the clinical significance of lactate and albumin, is developed and practically tested in several groups of patients (11–13). Results of these studies showed that LAR might be superior to single lactate in predicting mortality of critically ill patients. Moreover, recent research concluded that the prognostic value of LAR was better than single lactate on predicting neurologic outcomes and survival to discharge after out-of-hospital cardiac arrest (14). Therefore, we make a reasonable assumption that LAR is similarly valuable in predicting mortality of moderate to severe TBI patients. This observational study was designed to verify our scientific hypothesis.

## Materials and Methods

### Patients

This study was performed in a West China Hospital. Patients diagnosed with moderate to severe TBI and transferred to our hospital within 4 h after injuries between May 2015 and January 2018 were eligible in this study. Diagnoses of TBI were confirmed according to findings of CT and MRI. Exclusion criteria were listed as below: (1) patients transferred from other hospital after suffering injuries; (2) patients hospitalized in our hospital <48 h; (3) patients complicated with severe hepatorenal, cardiovascular, or respiratory diseases, cancer, and other central nervous system diseases; and (4) patients lacked in complete laboratory results. A total of 273 patients were finally included in this study. The study was approved by the ethics committee of West China hospital, Sichuan University. Informed consent forms for joining observational research of each patient were legally obtained from themselves or their authorized families when they were admitted based on the research policy of our hospital.

### Data Collection

Vital signs and Glasgow Coma Scale (GCS) were recorded once patients were admitted to the emergency department of our hospital. Injury Severity Scores (ISSs) of other regions except for the head were added. Sequential Organ Failure Assessment (SOFA) score on the first day of hospitalization was also recorded. The blood samples of patients in admission were taken for blood biochemistry, blood routine, and arterial gas analysis, such as lactate level. Laboratory results of the first blood sample on admission were recorded in this study for statistical analysis. Occurrence of hypoxia on admission was also collected and was divided into two severities, i.e., mild hypoxia (60 mmHg ≤ PaO_2_ <80 mmHg) and severe hypoxia (PaO_2_ <60 mmHg). The primary outcome of this study was in-hospital mortality.

### Statistical Analysis

Kolmogorov-Smirnov test was performed to test the normality of variables. Normally distributed variables were presented as mean ± SD, and non-normally distributed variables were presented as median (interquartile range). Moreover, categorical variables were shown in the form of numbers (percentage). Independent Student's *t*-test and Mann-Whitney *U*-test were respectively performed to analyze differences between two groups of normally distributed and non-normally distributed variables. We performed a Chi-square test to examine the difference of categorical variables. The relationship between LAR and other factors was tested by Spearman rank correlation analysis. Univariate and multivariate logistic regression were sequentially used to explore the association between risk factors and mortality in this study cohort. In addition, independent risk factors were incorporated to construct a prognostic model by multivariate logistic regression. We drew the receiver operating characteristic (ROC) curves and evaluated the predictive value of LAR and the prognostic model by calculating the area under the ROC curves (AUC). Z test was utilized to compare the difference of AUC between LAR and the prognostic model.

A value of *p* < 0.05 was considered to be of statistical significance. SPSS 22.0 Windows software (SPSS, Inc., Chicago, IL, USA) was used for all statistical analyses and figure drawing.

## Results

### Baseline Characteristics of Survivors and Non-survivors in TBI Patients

There were 124 survivors and 149 non-survivors with a mortality rate of 54.6% in this study (Table 1). Age and male ratio did not differ between survivors and non-survivors (43 vs. 43, *p* = 0.707; 77.4 vs. 73.8%, *p* = 0.491). Motor vehicle crash and falling injury respectively ranked first and second among the injury causes with 66.3 and 19.8%. Initial vital signs, such as systolic and diastolic blood pressure, heart rate, did not differ between survivors and non-survivors (123 vs. 120, *p* = 0.287; 72 vs. 70, *p* = 0.094; 98 vs. 103, *p* = 0.184). However, the body temperature of non-survivors was significantly lower than survivors (36.7 vs. 36.8, *p* = 0.003). In addition, non-survivors had significantly lower GCS than survivors (5 vs. 7, *p* < 0.001). Results of laboratory tests showed that non-survivors had a significant higher level of glucose, lactate, LAR, blood urea nitrogen (BUN), serum creatinine, lactate dehydrogenase (LDH), and prothrombin time (PT). Whereas the level of platelet, hemoglobin, and albumin was significantly lower in non-survivors. The incidence of subarachnoid hemorrhage (SAH), subdural hematoma (SDH), intraparenchymal hemorrhage (IPH), diffuse axonal injury (DAI), and cerebral infarction were both higher in non-survivors than survivors. In addition, non-survivors had shorter length of intensive care unit (ICU) stay and length of hospital stay than survivors (*p* < 0.001).

**Table 1:** Baseline characteristics of included patients.

**Variables**	**Full cohort (*N* = 273)**	**Survivors (*n* = 124, 45.4%)**	**Non-survivors (*n* = 149, 54.6%)**	***P* value**
Age (year)	43 (26–55)	43 (24–56)	43 (26–55)	0.707
Gender (male)	206 (75.5%)	96 (77.4%)	110 (73.8%)	0.491
Mechanism of injury				
Traffic accident	181 (66.3%)	78 (62.9%)	103 (69.1%)	0.279
High fall	54 (19.8%)	28 (22.6%)	26 (17.4%)	0.290
Stumble	26 (9.5%)	11 (8.9%)	15 (10.1%)	0.737
Others	12 (4.4%)	7 (5.6%)	5 (3.4%)	0.359
Vital signs in admission				
Systolic blood pressure (mmHg)	120 (106–138)	123 (108–138)	120 (103–137)	0.287
Diastolic blood pressure (mmHg)	71 (60–84)	72 (65–83)	70 (56–84.5)	0.094
Heart rate (bpm)	101 (82–120)	98 (81–117)	103 (84–123.5)	0.184
Body temperature (°C)	36.8 (36.5–37.1)	36.8 (36.5–37.5)	36.7 (36.3–37.0)	0.003
GCS in admission	5 (4–7)	7 (5–9)	5 (3–6)	<0.001
ISS _otherregions_	0 (0–5)	0 (0–8)	0 (0–4)	0.730
SOFA	6 (5–8)	6 (4–7)	7 (6–9)	<0.001
Hypoxia				0.595
None	233 (85.3%)	107 (86.3%)	126 (84.6%)	
Mild hypoxia	30 (11.0%)	14 (11.3%)	16 (10.7%)	
Severe hypoxia	10 (3.7%)	3 (2.4%)	7 (4.7%)	
Laboratory tests				
Glucose (mmol/L)	10.33 (7.87–14.29)	8.53 (6.61–11.59)	12.66 (9.16–16.15)	<0.001
White blood cell (10^9^/L)	15.13 (11.28–20.06)	15.09 (11.13–20.08)	15.47 (11.54–20.14)	0.512
Neutrophil (10^9^/L)	11.87 (8.67–15.24)	11.81 (8.96–15.43)	11.98 (7.81–15.12)	0.301
Lymphocyte (10^9^/L)	0.78 (0.53–1.12)	0.88 (0.54–1.23)	0.74 (0.50–1.04)	0.070
Platelet (10^9^/L)	90 (57–141)	114 (76–172)	72 (46–112)	<0.001
Hemoglobin (g/L)	85 (73–103)	92 (79–110)	81 (69–97)	<0.001
Albumin (g/dL)	3.02 (2.61–3.45)	3.25 (2.86–3.70)	2.77 (2.31–3.18)	<0.001
Lactate (mmol/L)	2.4 (1.5–3.6)	1.8 (1.2–2.8)	3.1 (2.1–4.6)	<0.001
LAR	0.78 (0.475–1.31)	0.5 (0.39–0.79)	1.09 (0.73–1.72)	<0.001
Blood urea nitrogen (mmol/L)	6.34 (4.81–8.82)	5.72 (4.36–7.69)	7.11 (5.35–9.91)	<0.001
Serum creatinine (umol/L)	76 (56–106)	65 (54–85)	84 (59–124)	<0.001
LDH (U/L)	400 (301–594)	360 (289–479)	452 (330–775)	<0.001
PT (s)	13.9 (12.5–16.3)	13.0 (11.9–14.6)	15.1 (13.4–18.2)	<0.001
Injury types				
Subarachnoid hemorrhage	142 (52.0%)	54 (43.5%)	88 (59.1%)	0.015
Epidural hematoma	27 (9.9%)	11 (8.9%)	16 (10.7%)	0.686
Subdural hematoma	88 (32.2%)	27 (21.8%)	61 (40.9%)	0.001
Intraparenchymal hemorrhage	164 (60.1%)	56 (45.2%)	108 (72.5%)	<0.001
Intraventricular hemorrhage	15 (5.5%)	6 (4.8%)	9 (6.0%)	0.792
Diffuse axonal injury	88 (32.2%)	30 (24.2%)	58 (38.9%)	0.013
Cerebral infarction	9 (3.3%)	1 (0.8%)	8 (5.4%)	0.043
Surgical interventions				
Decompressive craniectomy	102 (37.4%)	41 (33.1%)	61 (40.9%)	0.209
Hematoma evacuation	114 (41.8%)	54 (43.5%)	60 (40.3%)	0.623
Length of ICU stay (day)	10 (2–24)	21 (13–33)	2 (1–7)	<0.001
Length of hospital stay (day)	15 (5–34)	31 (22–48)	5 (3–12)	<0.001

*GCS, Glasgow Coma Scale; ISS, Injury Severity Score; SOFA, Sequential Organ Failure Assessment; LAR, lactate to albumin ratio; LDH, lactate dehydrogenase; PT, prothrombin time*.

### Correlation Between Other Factors and LAR by Spearman Analysis in Included TBI Patients

Spearman correlation analysis showed that GCS (*r* = −0.347, *p* < 0.001) and platelet (*r* = −0.444, *p* < 0.001) were negatively and moderately associated with LAR level, while SOFA (*r* = 0.443, *p* < 0.001), glucose (*r* = 0.447, *p* < 0.001), serum creatinine (*r* = 0.340, *p* < 0.001), and PT (*r* = 0.420, *p* < 0.001) were positively and moderately related with LAR level (Table 2).

**Table 2:** Correlation between other factors and LAR by Spearman analysis in patients with moderate to severe TBI.

**Factors**	***r***	***p***
Age (year)	–0.009	0.878
Gender (male)	0.022	0.714
Systolic blood pressure	−0.096	0.113
Diastolic blood pressure	−0.097	0.111
Heart rate	0.023	0.700
Body temperature	−0.175	**0.004**
GCS in admission	−0.347	**<0.001**
ISS _otherregions_	−0.051	0.401
SOFA	0.443	**<0.001**
Hypoxia	0.026	0.672
Glucose	0.447	**<0.001**
White blood cell	0.111	0.067
Neutrophil	−0.007	0.909
Lymphocyte	−0.094	0.121
Platelet	−0.444	**<0.001**
Hemoglobin	−0.235	**<0.001**
Blood urea nitrogen	0.106	0.082
Serum creatinine	0.340	**<0.001**
LDH	0.202	**0.001**
PT	0.420	**<0.001**
Subarachnoid hemorrhage	0.073	0.232
Epidural hematoma	0.124	**0.040**
Subdural hematoma	0.256	**<0.001**
Intraparenchymal hemorrhage	0.187	**0.002**
Intraventricular hemorrhage	−0.082	0.176
Diffuse axonal injury	−0.003	0.955
Cerebral infarction	0.116	0.056
Decompressive craniectomy	0.160	**0.008**
Hematoma evacuation	0.086	0.154

*TBI, traumatic brain injury; LAR, lactate to albumin ratio; GCS, Glasgow Coma Scale; ISS, Injury Severity Score; SOFA, Sequential Organ Failure Assessment. Bold values indicates p <0.05*.

### Univariate and Multivariate Analyses of Risk Factors for Mortality in Included TBI Patients

In univariate logistic regression analysis, we found that body temperature (OR = 0.674, *p* = 0.004), GCS (OR = 0.647, *p* < 0.001), platelet (OR = 0.993, *p* < 0.001), and hemoglobin (OR = 0.978, *p* < 0.001) were negatively correlated with outcomes in TBI patients (Table 3). Moreover, SOFA (OR = 1.475, *p* < 0.001), glucose (OR = 1.275, *p* < 0.001), LAR (OR = 3.611, *p* < 0.001), BUN (OR = 1.068, *p* = 0.030), serum creatinine (OR = 1.007, *p* = 0.003), LDH (OR = 1.002, *p* < 0.001), PT (OR = 1.292, *p* < 0.001), SAH (OR = 1.870, *p* = 0.011), SDH (OR = 2.490, *p* = 0.001), IPH (OR = 3.199, *p* < 0.001), and DAI (OR = 1.997, *p* = 0.010) were risk factors of mortality in the TBI patients. Furthermore, results of multivariate logistic regression analysis indicated that six factors, i.e., GCS (OR = 0.743, *p* = 0.001), glucose (OR = 1.132, *p* = 0.005), LAR (OR = 1.698, *p* = 0.022), SDH (OR = 2.889, *p* = 0.006), IPH (OR = 2.395, *p* = 0.014), and DAI (OR = 2.183, *p* = 0.041), were independently associated with mortality after adjusting confounders.

**Table 3:** Univariate and multivariate logistic regression analysis of risk factors for mortality in patients with moderate to severe TBI.

	**Unadjusted analysis**		**Adjusted analysis**	
**Variables**	**OR (95%Cl)**	***P* value**	**OR (95%Cl)**	***P* value**
Age (year)	1.003 (0.990–1.015)	0.683		
Gender (male)	0.823 (0.471–1.436)	0.492		
Systolic blood pressure	1.001 (0.997–1.005)	0.636		
Diastolic blood pressure	0.987 (0.973–1.001)	0.077		
Heart rate	1.006 (0.997–1.014)	0.176		
Body temperature	0.674 (0.514–0.883)	0.004	0.696 (0.482–1.006)	0.054
GCS in admission	0.647 (0.565–0.742)	<0.001	0.743 (0.621–0.888)	**0.001**
ISS _otherregions_	0.997 (0.960–1.035)	0.868		
SOFA	1.475 (1.291–1.686)	<0.001	1.001 (0.785–1.275)	0.996
Hypoxia		0.617		
None	1.000 [Reference]			
Mild hypoxia	0.971 (0.453–2.080)	0.939		
Severe hypoxia	1.981 (0.500–7.851)	0.330		
Glucose	1.275 (1.182–1.374)	<0.001	1.132 (1.037–1.235)	**0.005**
White blood cell	1.013 (0.979–1.048)	0.458		
Neutrophil	0.982 (0.942–1.024)	0.399		
Lymphocyte	0.680 (0.454–1.021)	0.063		
Platelet	0.993 (0.990–0.997)	<0.001	0.999 (0.995–1.004)	0.793
Hemoglobin	0.978 (0.967–0.990)	<0.001	0.996 (0.981–1.011)	0.604
LAR	3.611 (2.219–5.876)	<0.001	1.698 (1.078–2.675)	**0.022**
Blood urea nitrogen	1.068 (1.006–1.133)	0.030	1.001 (1.000–1.002)	0.185
Serum creatinine	1.007 (1.002–1.011)	0.003	1.114 (0.984–1.262)	0.089
LDH	1.002 (1.001–1.003)	<0.001	1.073 (0.990–1.163)	0.087
PT	1.292 (1.168–1.429)	<0.001	0.998 (0.991–1.005)	0.649
Subarachnoid hemorrhage	1.870 (1.154–3.029)	0.011	0.939 (0.471–1.873)	0.858
Epidural hematoma	1.236 (0.551–2.771)	0.607		
Subdural hematoma	2.490 (1.455–4.261)	0.001	2.889 (1.360–6.135)	**0.006**
Intraparenchymal hemorrhage	3.199 (1.932–5.297)	<0.001	2.395 (1.190–4.819)	**0.014**
Intraventricular hemorrhage	1.264 (0.437–3.655)	0.665		
Diffuse axonal injury	1.997 (1.179–3.382)	0.010	2.183 (1.034–4.610)	**0.041**
Cerebral infarction	6.979 (0.861–56.585)	0.069		
Decompressive craniectomy	1.403 (0.854–2.306)	0.181		
Hematoma evacuation	0.874 (0.539–1.416)	0.584		

*TBI, Traumatic brain injury; GCS, Glasgow Coma Scale; ISS, Injury Severity Score; SOFA, Sequential Organ Failure Assessment; LAR, Lactate to albumin ratio; LDH, Lactate dehydrogenase; PT, Prothrombin time. Bold values indicates p <0.05*.

### Predictive Value of LAR and Constructed Prognostic Model

**Figure 1:**
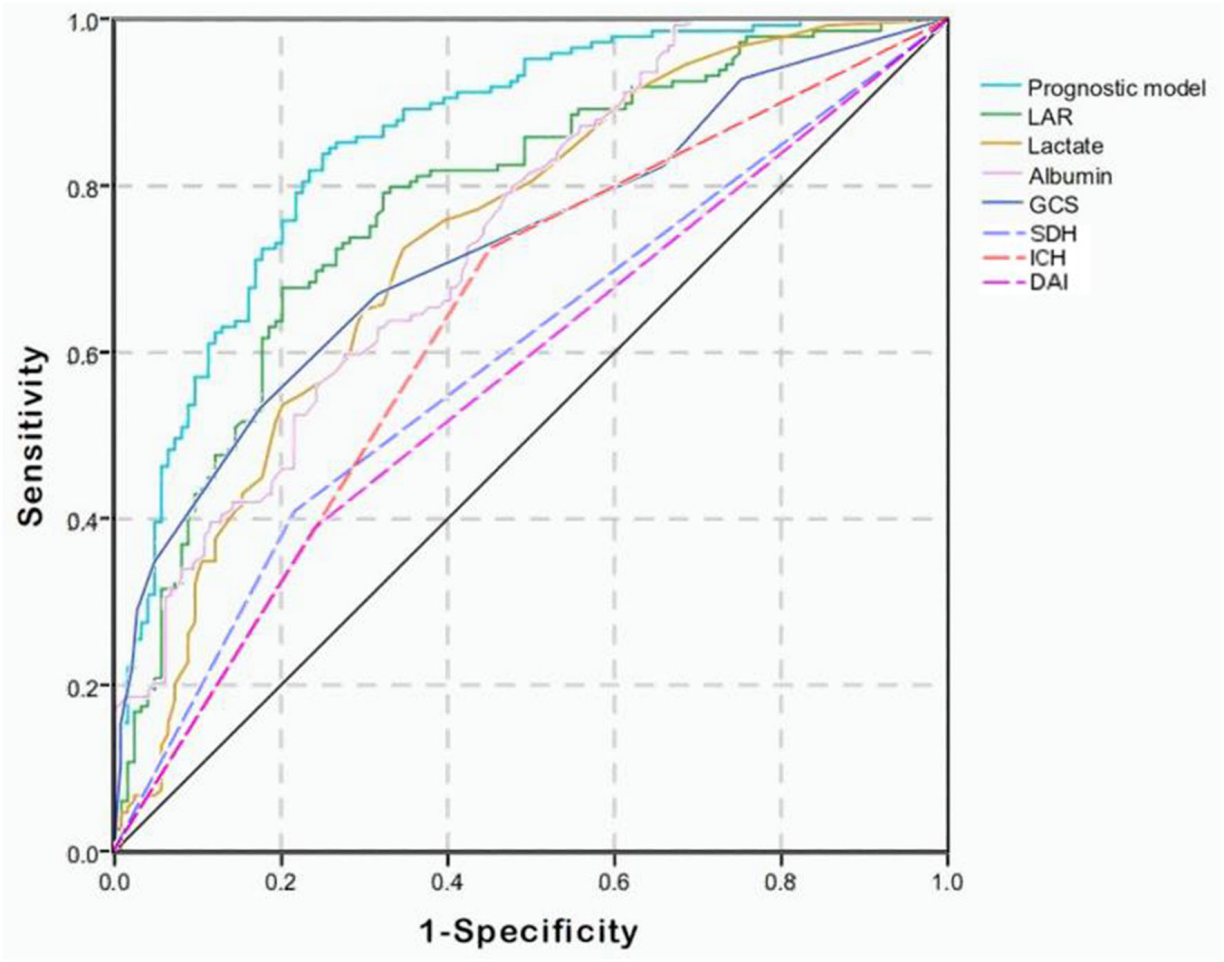
Receiver operating characteristic (ROC) curves of predictive factors and the prognostic model for predicting mortality in moderate to severe TBI patients. The area under the curve (AUC) of GCS, lactate, albumin, LAR, SDH, IPH, and DAI and the constructed prognostic model was 0.729, 0.733, 0.740, 0.780, 0.596, 0.637, 0.574, and 0.857, respectively.

Combining GCS, glucose, LAR, SDH, IPH, and DAI, we constructed a prognostic model for predicting mortality in the TBI patients by multivariate logistic regression analysis. As shown in Figure 1, the AUC values of single lactate, albumin, and LAR are 0.733 (95% Cl; 0.673–0.794), 0.740 (95% Cl; 0.683–0.797), and 0.780 (95% Cl; 0.725–0.835), respectively (Table 4). The AUC value of LAR and the prognostic model was 0.780 (95% Cl; 0.725–0.835) and 0.857 (95% Cl; 0.812–0.901), respectively. The AUC value of LAR was higher than that of GCS (Z = 1.221, *p* > 0.05), single lactate value (Z = 1.1251, *p* > 0.05), and single albumin (Z = 0.9923, *p* > 0.05) though without statistical significance. However, the prognostic model incorporating LAR had a significant higher AUC value than single LAR (Z = 2.1250, *p* < 0.05).

**Table 4:** Value of factors and constructed prognostic model on predicting mortality in patients with moderate to severe TBI.

**Predictive factors**	**AUC**	**95%Cl**	**Sensitivity**	**Specificity**
GCS	0.729	0.668–0.789	0.532	0.826
Lactate	0.733	0.673–0.794	0.725	0.653
Albumin	0.740	0.683–0.797	0.597	0.725
LAR	0.780	0.725–0.835	0.678	0.798
SDH	0.596	0.529–0.663	0.409	0.782
IPH	0.637	0.570–0.703	0.725	0.548
DAI	0.574	0.506–0.641	0.389	0.758
Prognostic model	0.857	0.812–0.901	0.839	0.750

*TBI, traumatic brain injury; AUC, area under the ROC curve; Cl, confidence interval; GCS, Glasgow Coma Scale; LAR, lactate to albumin ratio; SDH, subdural hematoma; IPH, intraparenchymal hemorrhage; DAI, diffuse axonal injury*.

*The prognostic model was composed of GCS, glucose, LAR, SDH, IPH, and DAI*.

## Discussion

Previous studies have shown the added prognostic value of lactate value in trauma patients (6, 15, 16). In addition, several research studies have confirmed that LAR was associated with mortality and the development of multiple organ dysfunction syndrome (MODS) in generalized or pediatric sepsis patients (11–13, 17, 18). A recent study showed that LAR had a higher value than single lactate level on predicting neurologic outcomes and survival to discharge in patients suffering out-of-hospital cardiac arrest (14). We make a reasonable hypothesis that LAR would also be superior to single lactate in predicting mortality in TBI patients.

As a component of LAR, the serum lactate is widely acknowledged as an indicator of inadequate tissue perfusion. In addition, the correlation between serum lactate and mortality has been verified in many clinical settings, such as sepsis, shock, and trauma (19–22). However, several studies exploring the association between lactate and outcome in TBI patients showed different conclusions (9, 23–25). One of these studies even indicated that TBI patients whose serum lactate >5 mmol/L were likely to have better survival than those with relatively low lactate level (24). Furthermore, the exogenous supplement of lactate by infusing hypertonic sodium lactate has been verified beneficial for survival and neurologic outcome and cognitive recovery in TBI animal models and patients (26–32). In our study, serum lactate was higher in non-survivors than survivors and was useful in predicting mortality in moderate to severe TBI patients with an AUC of 0.733. The most key point we think to understand and discuss the relationship between blood lactate and the outcome of TBI patients is the different meaning of increased serum lactate between the initial pathophysiological state and exogenous supplement state.

A previous study found that serum lactate would still increase even in normotensive TBI patients (24). This fact indicated an initial increase of serum lactate after TBI could not only be caused by peripheral tissue hypoperfusion due to blood loss, but also the worsening tissue oxygenation due to complications, such as acute lung injury and neurogenic lung edema. The detailed mechanism of initially increased lactate after TBI deserves further exploration. Initially being put forward in 1994, the astrocyte–neuron lactate shuttle has changed the opinion that lactate is only an useless waste during the anaerobic metabolism process (33). It was illustrated that astrocyte would uptake glucose and metabolized it into lactate under the stimulation of much glutamate. The generated lactate would be transferred to neurons and enter the tricarboxylic acid cycle for energy demand of brain. The increased serum lactate directly penetrating the blood-brain-barrier would also accumulate in neuronal intercellular space and be utilized by neurons for energy production (34, 35). Moreover, one study discovered that brain uptake of lactate reflected by arterio-venous differences for lactate (AVDlac) was higher in more severe TBI patients and non-survivors (36). Therefore, a reasonable conjecture is that uptake of lactate from neuron after more severe TBI would decrease more serum levels of lactate. However, it was confirmed that the magnitude of absorbed lactate by brain was extremely small compared with the magnitude of serum lactate level (36). Therefore, the initial fluctuation of serum lactate level after TBI is mainly attributable to pathophysiological changes of the systemic body but not of single brain. This argument might be confirmed by the finding of the previous study that blood lactate levels were associated with SOFA score, which reflects systemic organ failure in unspecified ICU patients (37). In addition, blood lactate level was also verified inversely associated with GCS in isolated TBI patients (24). Although higher serum lactate is beneficial for brain energy supplements, the effect of poor pathophysiologic condition indicated by higher serum lactate on outcome could be greater than relatively transient and small effect of energy supplement. Generally, the initially increased serum lactate in the natural pathophysiologic condition is inversely associated with favorable outcome in TBI patients by reflecting the degree of systemic organ failure and initial brain injury severity.

On the contrary, the continuously increased serum lactate level during exogenous infusion of hypertonic sodium lactate could indicate better survival and recovery after TBI (30, 38, 39). Because increased serum lactate during exogenous supplement is not a reflection of initial tissue hypoperfusion and organ failure, but only means more alternative energy fuel for the injured brain. This is a key point to distinguish the meaning of increased serum lactate under the initial pathophysiological condition and exogenous supplement condition. The beneficial effects of hypertonic sodium lactate on the injured brain have been definitely recognized, i.e., improving cerebral perfusion and brain glucose availability, reversing impaired brain metabolism, and oxygenation. (26–28). In addition to the function of neuroenergetic material, lactate is actually a crucial signaling molecule, which could modulate the production of pentose phosphate, an important molecule to prevent oxidative stress injury in brain (40–43). It was testified that lactate would provide 60% of the energy source for cerebral metabolism as blood lactate increases to 5 mmol/L (44, 45), To sum up, the increased serum lactate level during exogenous supplement of lactate is beneficial for neurologic and survival outcome and cognitive recovery after TBI.

The albumin level of non-survivors was significantly lower than survivors in this study. Produced by hepatocytes, albumin works in multiple ways to maintain the physiologic function of the healthy body, such as constituting plasma osmotic pressure, transporting insoluble small organic molecules, and combining heavy metal ions to eliminate their toxic effects. In addition, low albumin level is also considered as an efficient marker of malnutrition. The cause of hypoalbuminemia after TBI is diversified, i.e., initial blood loss due to injury, consumption by secondary oxidative stress injury, and physiological hypoalbuminemia resulted from massive crystal liquid infusion. The reduction of serum albumin and its association with mortality after TBI have been confirmed in previous studies (46–49). The correlation between hypoalbuminemia and poor outcome of TBI patients could be explained by the brain edema and subsequent increased intracranial pressure resulted from insufficient intravascular osmolality. In addition, a lower level of albumin could indicate more severe degree of the systemic inflammatory response, which was discovered correlated with poor outcome of TBI patients (50, 51). In our study, the AUC value of single lactate was 0.733. After the incorporation of albumin, the AUC value of LAR was increased to 0.780. This result indicated that LAR, calculated by the value of lactate and albumin, could more comprehensively reflect tissue injury severity and systemic organ function of TBI patients. The prognostic model constructed by us, which consisted of GCS, glucose, LAR, SDH, IPH, and DAI, is useful in predicting mortality of moderate to severe TBI patients with high discriminative ability and sensitivity.

This study had several limitations. Firstly, this observational study was performed in a single center so that the selection bias was inevitable. A further prospective study with a larger sample size in other centers should be conducted to externally validate the predictive value of our prognostic model. Secondly, the long-term neurologic outcome and recovery status were not followed up and the specific causes of death were not recorded so that we could not explore the correlation between LAR and them. Thirdly, the drugs and operations of prehospital emergency medical care which could influence the serum lactate level were not recorded by us. Our results might be confounded by these factors.

## Conclusion

The LAR is an effective and readily available marker of outcome in moderate to severe TBI patients. The prognostic model incorporating LAR with high predictive value is beneficial for clinicians to evaluate possible progression and make treatment decisions in moderate to severe TBI patients.

## Data Availability Statement

The raw data supporting the conclusions of this article will be made available by the authors, without undue reservation.

## Ethics Statement

The studies involving human participants were reviewed and approved by Ethics Committee of West China hospital, Sichuan University. The patients/participants provided their written informed consent to participate in this study.

## Author Contributions

RW: conception and design. MH: collection and assembly of data. RW and MH: data analysis and interpretation. RW and FQ: manuscript writing. JZ and JX: manuscript proofread and revision. All the authors take responsibility for the final manuscript and approve it for publication.

## Funding

This work was supported by the 1.3.5 Project for Disciplines of Excellence, West China Hospital, Sichuan University (ZYJC18007) and the Key Research and Development Project of Science and Technology Department of Sichuan Province (2019YFS0392).

## Conflict of Interest

The authors declare that the research was conducted in the absence of any commercial or financial relationships that could be construed as a potential conflict of interest.

## Publisher's Note

All claims expressed in this article are solely those of the authors and do not necessarily represent those of their affiliated organizations, or those of the publisher, the editors and the reviewers. Any product that may be evaluated in this article, or claim that may be made by its manufacturer, is not guaranteed or endorsed by the publisher.
